# Lacunes are associated with late-stage multiple sclerosis comorbidities

**DOI:** 10.3389/fneur.2023.1224748

**Published:** 2023-08-08

**Authors:** Lijie Zhang, Xintong Yu, Yexiang Zheng, Aiyu Lin, Zaiqiang Zhang, Shaowu Li, Ning Wang, Ying Fu

**Affiliations:** ^1^Department of Neurology and Institute of Neurology of The First Affiliated Hospital, Institute of Neuroscience, Fuzhou, China; ^2^Fujian Key Laboratory of Molecular Neurology, Fujian Medical University, Fuzhou, China; ^3^Department of Neurology, China National Clinical Research Center for Neurological Diseases, Beijing Tiantan Hospital, Capital Medical University, Beijing, China; ^4^Department of Neuroimaging, Beijing Neurosurgical Institute, Beijing Tiantan Hospital, Capital Medical University, Beijing, China

**Keywords:** multiple sclerosis (MS), cerebral small vessel disease (CSVD), lacune, disease duration, arteriolar damage

## Abstract

Multiple sclerosis (MS) is a condition that affects the veins and small blood vessels. Previous research suggests that individuals with MS have an increased risk of vascular events and higher mortality rates. However, the relationship between MS and cerebral small vessel disease (CSVD) remains uncertain. This study aims to investigate the association between MS and lacunes. A prospective observational study was conducted, including a total of 112 participants, of which 46 had MS and 66 had CSVD. All participants underwent an MRI scan and a battery of neurological functional assessments. The presence of definite lacunes and black holes was determined through the analysis of T2-weighted, T1-weighted, and FLAIR images. The occurrence of lacunes in MS patients was found to be 19.6%. Notably, the duration of MS was identified as the sole risk factor for the development of lacune lesions in MS patients [odds ratio (OR) = 1.3, 95% confidence interval (CI) = 1.1–1.6, *p* = 0.008]. Comparatively, MS patients with lacunes exhibited a higher frequency of attacks and larger volumes of T2 lesions compared to MS patients without lacunes. Further analysis using receiver operating characteristic (ROC) curves showed that lacune lesions had limited ability to discriminate between MS and CSVD when disease duration exceeded 6 years. The presence of small arterial lesions in the brain of individuals with MS, along with the duration of the disease, contributes to the development of lacunes in MS patients.

## Introduction

Black holes in multiple sclerosis (MS) are characterized by T1 hypointense and T2 hyperintense lesions on MRI (1). These lesions can be categorized as either acute black holes, which may either progress to permanent black holes or transform into transient black holes (2). The exact etiology and pathogenesis of black holes remain uncertain. Some studies have proposed that CD8-mediated immune damage may contribute to the formation of black holes (3–5).

While MS primarily affects veins and venules, cerebral small vessel disease (CSVD) primarily affects small arteries. Aging, vascular risk factors (VRFs), and chronic inflammation are known to cause damage to the microvascular system, including the small arteries, resulting in hypoperfusion and tissue hypoxia that can influence the extent and distribution of MS-related pathology (6).

Lacunes, which are residual lesions of lacunar infarction or small hemorrhagic foci, serve as prominent imaging features of CSVD, reflecting intracranial arteriole lesions (7). Therefore, in this study, we focused on monitoring lacunar lesions to explore potential associations between CSVD and MS. We aimed to investigate the incidence of lacunes in an MS cohort and evaluate the related risk factors based on demographic and clinical characteristics, shedding light on the potential clinical significance of lacunes in MS patients.

## Materials and methods

### Patients

In this ongoing study, we prospectively collected clinical, demographic, and imaging data from patients diagnosed with MS and CSVD. The patients were hospitalized at Tian Tan Hospital of Capital Medical University in Beijing, China, between 2015 and 2017. MS diagnosis was made according to the 2010 McDonald MS standard (8) while CSVD diagnosis followed the 2013 European “Neuroimaging standards for research into small vessel disease” (9). Patients who fell into both categories of MS and CSVD were explicitly excluded from this study. All participants completed a structured questionnaire (10) and underwent physical and neurological assessments during their hospital stay (11).

Criteria for patient selection in our investigation were as follows:

1. Undergoing an MRI examination at least 2 days after the completion of physical and/or neurological examinations during hospitalization.
2. Having a fully documented medical record including information on hypertension, hyperlipidemia, diabetes, atrial fibrillation, current smoking, alcohol use, age at onset, age at baseline, disease duration at baseline, number of attacks, annual recurrence rate (ARR), number of relapses, and the use of any form of disease-modifying therapy (DMT).
3. Undergoing a comprehensive neurological examination, including the Expanded Disability Status Scale (EDSS) and modified Rankin scale (mRS).

Patients were excluded based on the following criteria: (a) MS patients who experienced relapse and receive steroid treatment within 30 days before study entry, (b) Pre-existing medical conditions associated with brain pathologic processes such as cerebrovascular disease or a history of alcohol abuse, (c) Confirmation of ischemic or hemorrhagic infarcts in the brain as observed in MRI, and (d) Pregnancy.

### MRI protocol

All patients underwent brain MRI using a 3.0-T scanner (Magnetom Trio Tim; Siemens) equipped with a 32-channel head coil. The imaging protocol included diffusion-weighted imaging (DWI), T2-weighted imaging, fluid-attenuated inversion recovery (FLAIR), and T1-weighted imaging (12).

### MR imaging analysis

**Figure 1:**
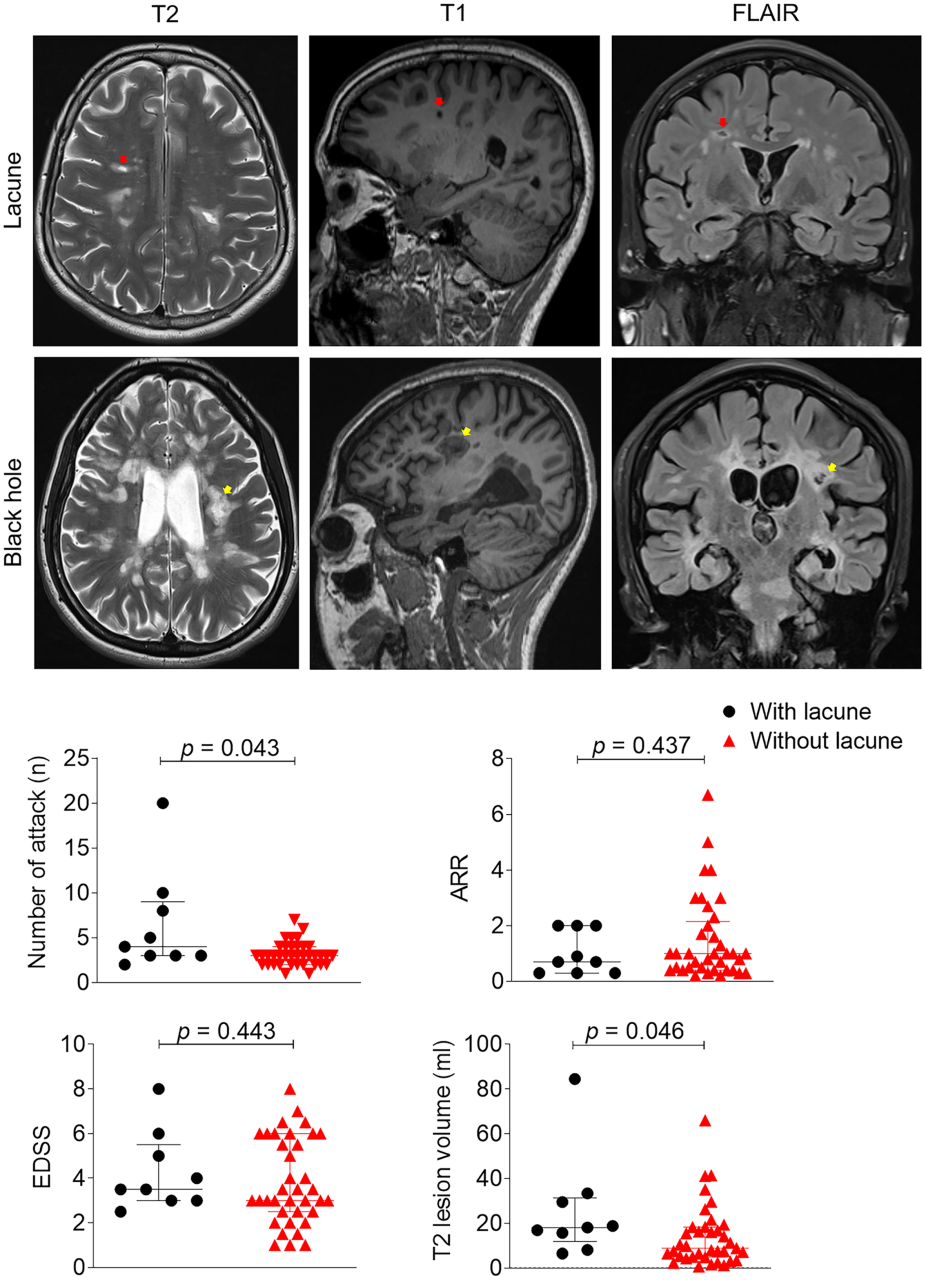
Clinical and imaging differences between patients with or without lacune. Example of lacune (red arrow) and black hole lesion (yellow arrow) in this study: Lacunas defined as round or ovoid, subcortical, fluid-filled cavities 3–15 mm in diameter in the territory of 1 perforating arteriole, specifically, lesions were round or elliptical in shape, and edges were neat, showing a liquid or near-liquid low signal on the T1 image. EDSS, expanded disability status scale, ARR, annual relapse rate; inferrential statistical analysis was performed with Mann–Whitney tests.

During the analysis, the MR imaging experts were blinded to the physical and neurological conditions of the subjects. Two experienced neuroimagers, also blinded to MR images obtained through other analytical sequences, assessed lacune lesions on T2-, T1-weighted, and FLAIR images. Lacunes were defined as a round or ovoid subcortical fluid-filled cavity (CSF-like signal) between 3 mm and approximately 15 mm in diameter, consistent with a previous acute small subcortical infarct or haemorrhage in the territory of a perforating arteriole. The diameter of the involved perforating arteriole varies from 40 to 850 μm, and the diameter of the associated infarct ranges from 2 to 3 mm to 15 mm or larger (9). Importantly, we distinguished these lesions from “black holes” (13, 14), which were round or elliptical in shape with neat edges and showed a liquid or near-liquid low signal on the T1 image. In addition, there is rarely a low signal on the FLAIR image due to the presence of myelin regeneration in the lesion. T2-, T1-weighted, and FLAIR images (Figure 1) were used to identify definite lacune lesions and black holes. To determine the volumes of individual white matter hyperintensity (WMH) lesions, manual segmentation was performed using MRIcro software based on the T2 images.^1^

### Reproducibility of lacune assessment

To ensure accuracy in identifying lacune lesions, two raters independently assessed their presence. Both raters were blinded to each other’s evaluations and their own previous assessments. Interclass correlation coefficients were calculated, and the values exceeded 0.8. Where there was discrepancy between the two raters, a third rater’s assessment was used.

### Statistical analysis

All statistical analyses were conducted using SPSS 17.0 (SPSS Inc., Chicago, IL). Differences between MS patients with and without lacune lesions were assessed using chi-square and Mann–Whitney tests. We used chi-square tests for gender, hypertension, hyperlipidemia, diabetes, atrial fibrillation, current smoking, and alcohol between MS patients with and without lacune lesions, as well as to compare the frequency of lacune in different groups of patients. Binary logistic regression analysis was performed to adjust for potential confounding factors. The diagnostic value of lacune lesions was determined using receiver operating characteristic (ROC) curves, with stratified evaluation at a time point of 6 years.

## Results

We enrolled a total of 46 patients with MS and 66 patients with CSVD through a meticulous selection process. Among the MS patients, the median age was 32 years (range: 25–56 years), and the median disease duration was 5 years (range: 2–7 years). Fifteen (33%) of the MS patients were male (Table 1). Nine out of the 46 MS patients met the imaging threshold for lacune lesions (19.6%) (Supplementary Figure S1). We performed Mann–Whitney tests to examine the clinical and demographic characteristics of patients with and without lacune lesions. No significant differences were observed in age or vascular risk factors between the two groups (Table 1). However, there was a significant difference in disease duration [median (IQR), 4 (1.8–6.0) vs. 10 (4.3–13.5), *p* = 0.016]. To adjust for age, we conducted binary logistic regression analysis, which revealed a significant association between disease duration (OR = 1.3, 95% CI = 1.1–1.6, *p* = 0.008) and the presence of lacune lesions. MS patients with lacune lesions exhibited a higher frequency of attacks (6 vs. 3, *p* = 0.043) and larger T2 lesion volumes (19 vs. 23 mL, *p* = 0.046) compared to those without lacune lesions (Figure 1). No differences were observed in ARR (0.7 vs. 1.0, *p* = 0.437), EDSS (3.5 vs. 3.0, *p* = 0.443) and mRS (1.0 vs. 1.0, *p* = 0.482).

**Table 1:** Demographic and clinical characteristics of patients.

Characteristic	MS without lacuna (*N* = 37)	MS with lacuna (*N* = 9)	*P1-value*	MS (*N* = 46)	CSVD (*N* = 66)	*P2-value*
Age, year, median (IQR)	29 (24–36)	36 (29–52)	0.074	32 (25–36)	54 (47–61)	<0.001
Male sex, *n* (%)	12 (32)	3 (33)	1.000	15 (33)	31 (47)	0.129
Hypertension, *n* (%)	0 (0)	1 (11.1)	0.196	1 (2)	42 (64)	<0.001
SBP, mmHg, median (IQR)	115 (105–120)	120 (113–123)	0.233	115 (109–120)	133 (122–144)	<0.001
DBP, mmHg, median (IQR)	75 (67–80)	75 (70–80)	0.387	75 (70–80)	80 (73–86)	<0.001
Hyperlipidemia, *n* (%)	7 (19)	4 (44)	0.24	11 (24)	30 (46)	0.020
Diabetes, *n* (%)	1 (2.7)	0 (0)	1.000	1 (2)	11 (17)	0.033
Atrial fibrillation, *n* (%)	0 (2.7)	0 (0)	1.000	0 (0)	1 (2)	1.000
Current smoking, *n* (%)	1 (2.7)	0 (0)	1.000	1 (2)	23 (35)	<0.001
Alcohol, *n* (%)	1 (2.7)	0 (0)	1.000	1 (2%)	17 (26)	0.001
Disease duration, year, (median, IQR)	4 (1.8–6.0)	10 (4.3–13.5)	0.016	5 (2–7)	2 (1–5)	0.001

Hypertension, systolic blood pressure > 140 mmHg and/or diastolic blood pressure > 90 mmHg; Diabetes, fasting plasma glucose ≥ 7.0 millimoles per liter and/or plasma glucose after a meal for two hours ≥ 11.1 millimoles per liter; Hyperlipidemia, serum total cholesterol levels > 5.72 millimoles per liter and/or triglycerides > 1.7 millimoles per liter. MS, multiple sclerosis; CSVD, cerebral small vessel disease; IQR, inter quartile range. *χ*^2^ test and Mann–Whitney test for differences between patient with lacuna and without (*P1*) or for differences between CSVD patient and MS patient (*P2*).

**Figure 2:**
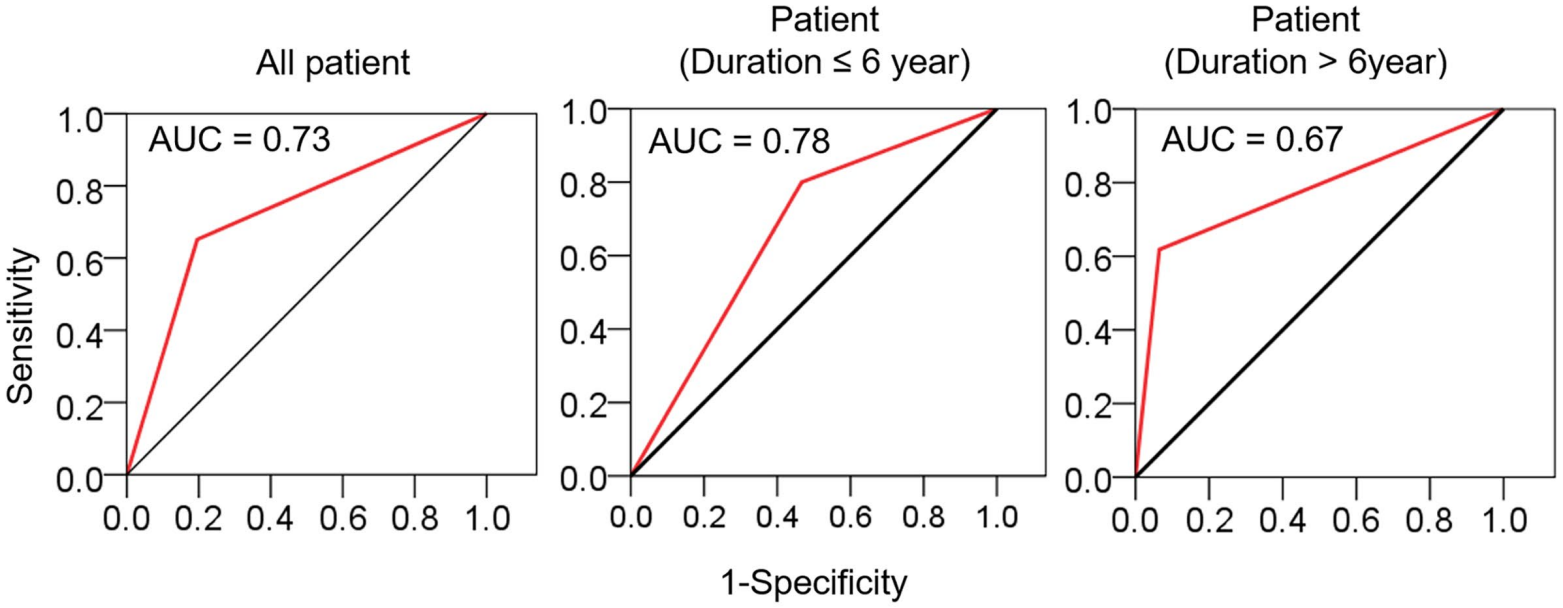
The sensitivity and specificity of lacune in distinguishing MS from CSVD. ROC curves were used to evaluate the diagnostic value of lacune, and the stratified evaluation was performed at a time point of 6 years. AUC, area under curve.

Comparison between the MS and CSVD groups revealed a higher incidence of traditional VRFs among CSVD patients (Table 1). There was a significant difference in the incidence of lacunes between the CSVD and MS groups (65.1% vs. 19.6%, *p* < 0.001), with an area under the ROC curve (AUC) of 0.73. However, when stratified by disease duration at a time point of 6 years, the AUC values decreased for patients with a disease course of >6 years (AUC = 0.67) (Figure 2), suggesting a diminishing ability to discriminate lacunar lesions with prolonged disease duration. Notably, the frequency of lacune lesions in MS patients with a disease duration over 6 years was comparable to that of CSVD patients (46.7 vs. 65.1%, *p* = 0.184).

## Discussion

Our study aimed to investigate the frequency and clinical significance of lacune lesions in individuals diagnosed with MS. By comparing the clinical features of MS patients with and without such lesions and analyzing the ROC curve after age stratification, we found that disease duration was the only significant risk factor for the occurrence of lacune lesions in MS patient. Furthermore, these findings suggest that the duration of MS increases the risk of CSVD, highlighting the time sensitivity and the importance of initiating preventive treatments at an appropriate time.

To the best of our knowledge, there are no existing reports exploring the potential relationship between lacune lesions and MS. Classical descriptions of lacunae can include lacune lesions (9), which can be challenging to distinguish from “black holes” in images (13, 14). To overcome this challenge, we further defined the distinguishing characteristics of lacune lesions in the imaging data of our study cohort. Based on our assessment criteria, we determined that the incidence of lacune lesions in the study population was 19.6%. It is important to note that the actual incidence is expected to be higher than the reported value we detected.

Traditional VRFs such as hypertension, diabetes, hyperlipidemia, and smoking were not significant factors in MS patients (with incidence rates of 2.2%, 2.2%, 23.9%, and 2.2% respectively). This could be attributed to the younger age [median (IQR): 31.5 (24.8–36.3)] of the enrolled population in our study compared to the aforementioned study. Several publications have shown an increased rate of ischemic stroke (IS) in MS patients (15, 16). Similarly, CSVD patients also exhibit a higher incidence of IS. Therefore, we were interested in exploring the differences in the development and causal influences of lacunes in MS patients compared to CSVD patients. The level of VRFs was higher in the CSVD cohort compared to the MS cohort, and the overall incidence of lacunes was also higher. When drawing ROC curves using lacune lesions, age-stratified analysis revealed that when the disease duration exceeded 6 years, lacune lesions had limited discriminatory capability between MS and CSVD. This further emphasizes the association between lacune lesions and MS disease duration, suggesting that intervention therapy for CSVD should be initiated at an appropriate time in the MS population.

There are several limitations to our study that should be acknowledged. First, the sample size was relatively small and should be expanded to further support our conclusions. Additionally, the mechanism underlying the increased risk of cerebral small vessel disease in MS patients remains unclear. It is hypothesized that MS disease creates a chronic inflammatory environment that damages small arterioles in the brain (6), but empirical confirmation is needed.

## Data availability statement

The original contributions presented in the study are included in the article/Supplementary material, further inquiries can be directed to the corresponding authors.

## Ethics statement

The studies involving human participants were reviewed and approved by Tian Tan Hospital of Capital Medical University. The patients/participants provided their written informed consent to participate in this study.

## Author contributions

YF formulated the study concept. YF and AL acquired funding for the study. YF and NW designed the study. LZ, YZ, ZZ, XY, and SL collected data. SL, YF, LZ, and YZ analyzed the data. YF and YZ wrote the manuscript. All authors contributed to the article and approved the submitted version.

## Funding

This work was supported by the grants U21A20360 (YF) and 82271375 (AL) from the National Natural Science Foundation of China; the grant 2020Y9129 (YF) from the Joint Funds for the innovation of science and Technology of Fujian Province.

## Conflict of interest

The authors declare that the research was conducted in the absence of any commercial or financial relationships that could be construed as a potential conflict of interest.

## Publisher’s note

All claims expressed in this article are solely those of the authors and do not necessarily represent those of their affiliated organizations, or those of the publisher, the editors and the reviewers. Any product that may be evaluated in this article, or claim that may be made by its manufacturer, is not guaranteed or endorsed by the publisher.

## Footnotes

^1^http://www.cabiatl.com/mricro/mricro/
