# Supplementary material for: Lacunes are associated with late-stage multiple sclerosis comorbidities
Source: Front Neurol. 2023 Aug 8;14:1224748. doi: 10.3389/fneur.2023.1224748 (PMC10442480; doi:10.3389/fneur.2023.1224748)

Supplementary material

Supplementary Figure 1. The representation of lacunes in MS and CSVD patients. The largest lacune in MS was shown in patient 1 to patient 9 (red arrow), and patient 10 showed a typical lacune in CSVD (blue arrow). Especially, patient 6 also showed black hole lesion (yellow arrow). MS = multiple sclerosis; CSVD = cerebral small vessel disease.

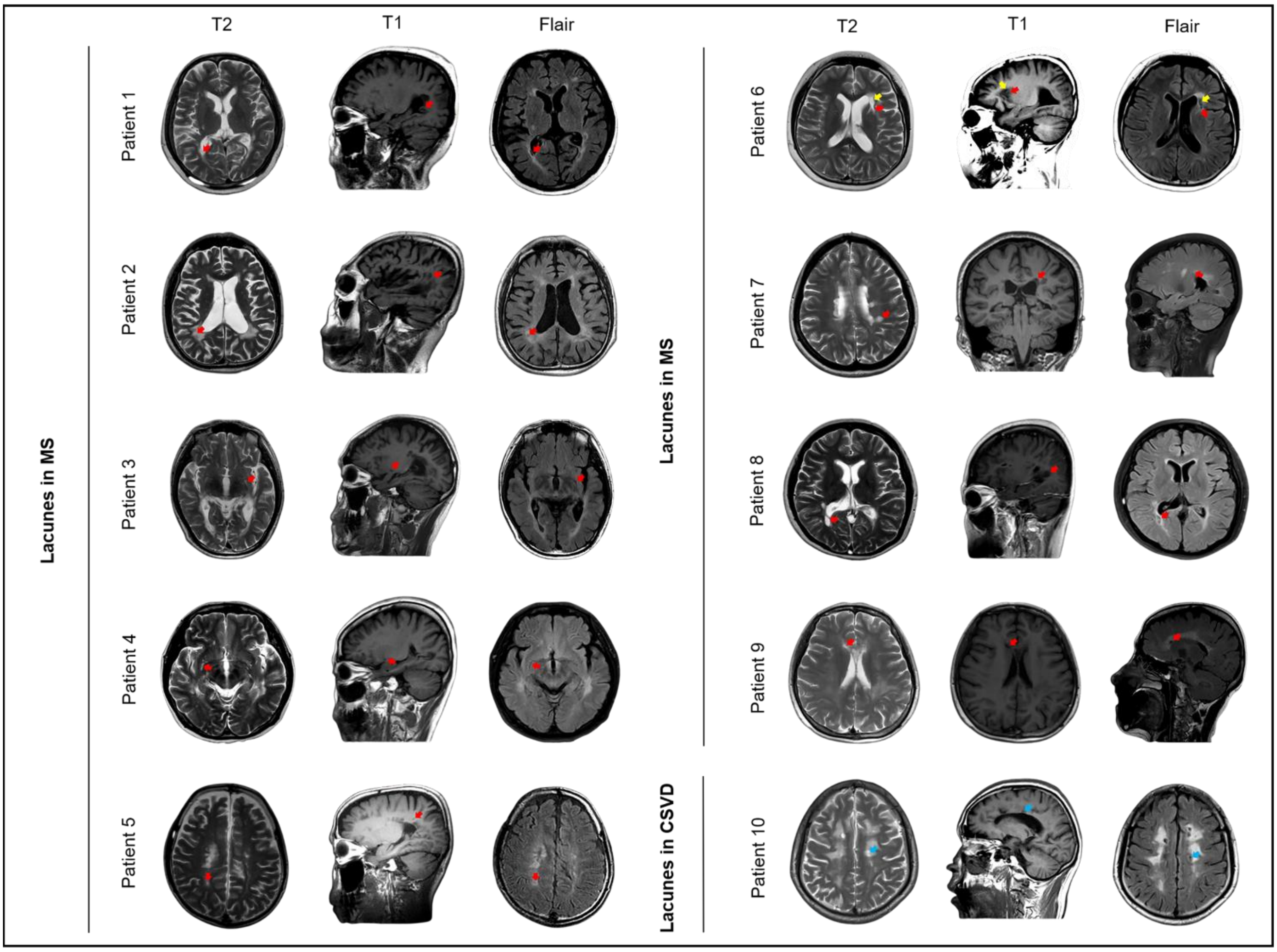

Supplement: Supplementary file 1 [file Image_1.PDF]
